# The Rat Genome Database Curators: Who, What, Where, Why

**DOI:** 10.1371/journal.pcbi.1000582

**Published:** 2009-11-26

**Authors:** Mary Shimoyama, G. Thomas Hayman, Stanley J. F. Laulederkind, Rajni Nigam, Timothy F. Lowry, Victoria Petri, Jennifer R. Smith, Shur-Jen Wang, Diane H. Munzenmaier, Melinda R. Dwinell, Simon N. Twigger, Howard J. Jacob, the RGD Team

**Affiliations:** Rat Genome Database, Human and Molecular Genetics Center, Medical College of Wisconsin, Milwaukee, Wisconsin, United States of America; University of California San Diego, United States of America

Biological databases have become ubiquitous as demonstrated by the 1,170 molecular biology databases catalogued in the 2009 Nucleic Acids Research online Molecular Biology Database Collection [1]. While researchers have come to rely on the valuable data in these resources, there often is little understanding of how the data are acquired and integrated, and what roles professional curators play in these processes. The increasing number of curators, their importance to the research world, and growing recognition of their professional contributions have resulted in two important events this year: the official creation of the International Society for Biocuration [2] and the launch of DATABASE–The Journal of Biological Databases and Curation by Oxford Journals [3]. These will provide valuable professional platforms to publish the research, data developments, and other advances created by thousands of biocurators around the world and to educate the public about this vital, developing profession. One database where such curation work takes place is the Rat Genome Database (RGD; http://rgd.mcw.edu) [4].

Established in 1999, RGD is the premier repository of rat genomic and genetic data and currently houses over 33,421 rat genes as well as human and mouse homologs, 1,698 rat (all that have been published to date) and 1,579 human quantitative trait loci (QTL), and 2,114 rat strains. Biological information curated for these data elements includes disease associations, phenotype data, pathways, molecular functions, biological processes, and cellular components. Curators are involved in acquiring and validating data elements, attaching biological information to elements, identifying pathways, and making connections among data types. The following glimpse at the curators and curation processes at RGD is designed to illustrate who curators are, what they do, where their results can be seen, and why their efforts make researchers' lives easier.

## Who Are the Curators?

Currently, RGD has seven full-time curators who bring diverse educational specialties and research expertise to the curation process. Five of the curators have PhDs and two have master's degrees. Educational specialties include molecular and cellular biology, physiology, biochemistry, microbiology, experimental pathology, and organic chemistry. As is common in the field of curation, the RGD curators bring a vast amount of experience in wet lab research ranging from 9 to 26 years with a mean of 16.9 years. They are well versed in research methods for cell and tissue culture, protein biochemistry, molecular biology, large and small animal physiology and surgery, developmental biology, infectious disease, microbiology, virology, biophysics, and bio-computation. Because of the wide array of experiences of curators, RGD developed a comprehensive training program and curation manual to ensure that curators follow rigorous standards in identifying data elements, assigning nomenclature, and annotating biological information to genes, QTLs, and strains. RGD adheres to the Gene Ontology (GO) (www.geneontology.org) guidelines and has adapted similar guidelines for other types of biological information. Biological annotations are based on experimental results published in peer-reviewed journals. New curators are trained by a senior curator and do test curation projects for several months before curating independently. Curators begin with a single data type, such as gene curation, and a single type of ontology-based biological annotation in order to develop adequate skills in this area before training in other data types. In addition to the curation manual and one-on-one training, there is a weekly curation meeting in which standards, policies, and new data types are discussed in order to maintain existing standards and develop new ones. Standards for new data types are developed through consultations with researchers having expertise in those areas, other model organism database groups, and the curation group at RGD. Inter-curator disagreements, which do occur even between highly experienced curators, are discussed in weekly meetings with pertinent examples from the literature and, when necessary, consultation with outside experts. Consensus decisions are added to the curation manual. There are additional mechanisms through which curators can submit new terms to specific ontologies, often hosted at SourceForge to accommodate data not covered by current procedures. Periodically all curators at RGD curate the same paper and these results are compared and discussed in the meeting to ensure consistency across curators. It takes approximately 1 year for a curator to become competent in curating one area of data and several years for real expertise to develop. While two of RGD's curators are new to the field, the other five have between 2 and 8.5 years of curation experience with an average of 5.7 years. Traits that RGD curators cite as valuable in being a curator include broad knowledge of scientific research and research methods, attention to detail, perseverance, and the ability to collaborate as a group. Many point out that curation is a rewarding alternative career for scientists to one in bench research. While curators are trained to serve in multiple roles and handle various types of data, they do specialize in particular areas to promote standardization and facilitate acquisition of greater knowledge and expertise. Following is a discussion of curation and other functions performed by RGD curators with an emphasis on *what* is done, *where* these results can be seen, and *why* these efforts are important to researchers.

## What Curators Do and Where Their Work Can Be Accessed

### Genomic Element Identification–Pipelines

**Figure 1:**
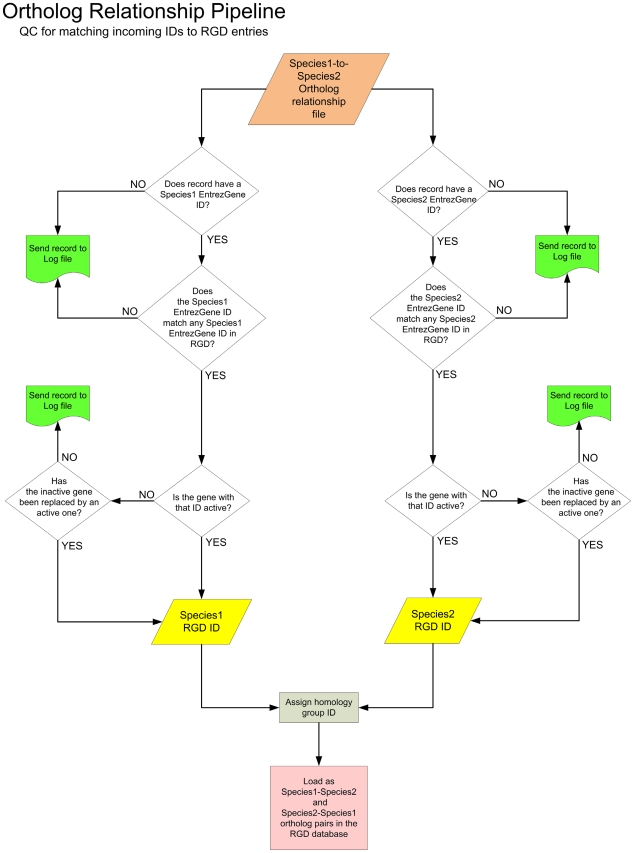
Quality Control (QC) for orthology relationship pipeline. Overview of the automated pipeline decision-making process used to generate relationships between rat, human, and mouse genes.

Curators play an important role in the identification and verification of genomic elements such as genes and QTLs, and of the multiple types of rat strains used in research today. While many are familiar with the role curators play in extracting data from the literature, less understood is the role they play in automated data acquisition processes. At RGD, a number of automated pipelines are used for data acquisition from other databases and for quality control. Examples include pipelines to import basic rat, mouse, and human gene and sequence data from the searchable gene database Entrez Gene, and one which imports an orthology relationship dataset shared by RGD, Mouse Genome Informatics (MGI), and the HUGO (Human Genome Organization) Gene Nomenclature Committee (HGNC; Figure 1). Other pipelines bring in identifiers and links to genes and proteins from the International Protein Index (IPI) and Ensemble, among others. Curators identify the data sources, design the quality control measures to be included in the pipeline, and test the pipeline output for validation. In addition, each weekly run of the pipeline creates a file of data conflicts which is reviewed by a curator with the conflicts being resolved manually.

**Figure 2:**
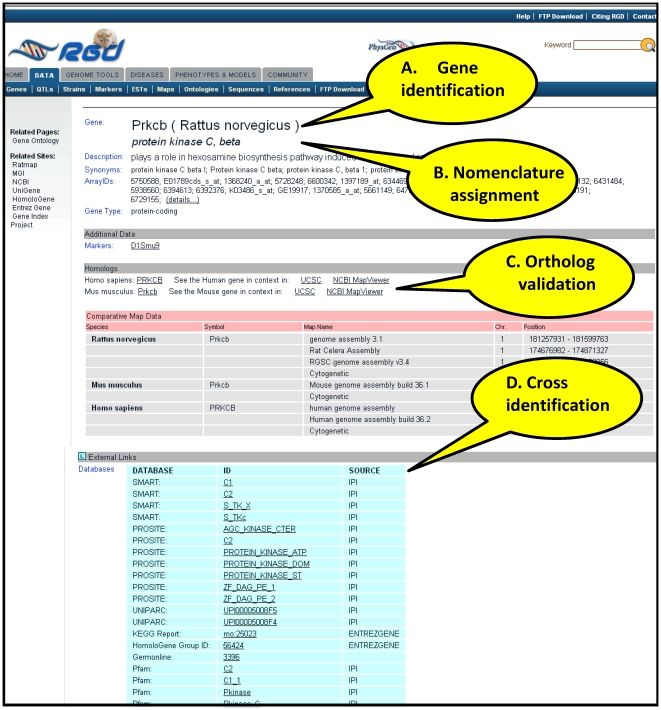
Gene report page showing curator contributions to gene identification and verification.

Approximately 1,500 gene conflicts are resolved annually as more sequence becomes available, gene models are refined, and orthology relationships reviewed and validated. These comprehensive procedures ensure proper gene identification, presented on gene report pages available to users on the RGD Web site (Figure 2A), and allow the curator to assign official nomenclature (Figure 2B), proper orthology relationships (Figure 2C), and provide accurate alignment with data at other sources (Figure 2D). Gene identification and nomenclature are exchanged with resources such as Entrez Gene, IPI, Mouse Genome Database (MGD), HGNC, and others to provide a shared accurate rat gene dataset. This allows researchers to retrieve the same genes of interest at multiple sites, to look at the genes across species, and to navigate from RGD to outside resources with confidence that they are reviewing the same genes. Because the same gene may have been referred to by a variety of symbols and names over the years, and may have multiple sequences and identifiers attached to it, it would be extremely difficult for researchers to determine whether a gene referenced in a paper or at a database is identical to one named elsewhere without the efforts by RGD curators to validate identification and ensure accuracy.

### QTL and Strain Identification

QTLs and strains are generally extracted from the literature or are submitted to RGD by researchers. Using data submission forms on the RGD Web site, researchers specify whether the work they are submitting is published or not. If the latter is the case, they can elect to have their submitted data displayed only after its publication. Credit is given for data displayed prior to publication as a reference indicating data submitted via personal communication by the researcher to RGD. After publication, credit is given on the report page as the cited reference containing the data. As with genes, the curators ensure identity and determine definitions and descriptions for QTLs and strains, as well as assign nomenclature. Curators record the flanking and peak QTL markers to ensure accurate genomic positions, and indicate the associated traits, as well as the strains used in the cross. This provides an unambiguous identity for each QTL, allowing researchers to view QTLs on map tools and to differentiate one QTL from another knowing with confidence whether a QTL referenced in one paper is the same one mentioned in another paper. Curators also provide nomenclature and history for strains and substrains to identify the correct genetic background. As next generation sequencing makes it possible to produce genome sequences for individual strains, this accurate tracking of strains and their history will become more important as researchers attempt to link phenotype data from multiple studies with the correct genome data.

### Biological Data Curation

**Figure 3:**
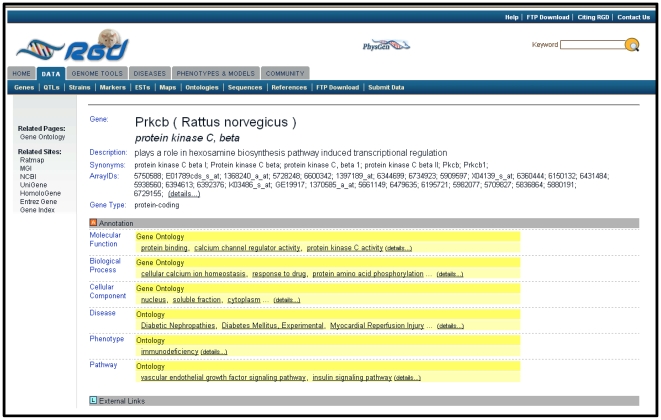
Curated biological annotations for Gene Ontology, diseases, phenotypes, and pathways.

Providing comprehensive biological information on rat genes, QTLs, and strains is a formidable task both in number of elements to be curated and the amount of literature available. Of the more than 35,400 genes in RGD, 31,304 have associated sequence data or RefSeqs, and of these, 15,607 are protein-coding with curated RefSeqs [4],[5]. In addition there are 2,114 strains with approximately 200 added per year and 1,698 QTLs with over 180 added per year. A search at PubMed (http://www.ncbi.nlm.nih.gov/Literature/) reveals there are over 1,267,000 rat manuscripts. In order to provide functional information for as many genomic elements and strains as possible and to leverage the information in the vast amount of rat literature, RGD has implemented a number of processes. RGD uses four ontologies to standardize biological information for genes, QTLs, and strains. These include the GO [6], the Mammalian Phenotype Ontology (MP) [7], a disease ontology (DO) based on the Medical Subject Headings (MeSH) [8], and the Pathway Ontology developed at RGD (PW) (Figure 3) [4]. In general, RGD's primary focus is manual annotation of rat genes, while GO annotations for mouse and human orthologs are brought in from the MGD and the Gene Ontology Annotation Database (GOA), respectively. Disease and pathway annotations, however, are added to the mouse and human orthologs during the manual curation process. Biological annotations consist of several parts including the ontology term, the evidence code, and the reference. Evidence codes for the GO indicate that the annotation is based on experimental results, direct assays, physical interactions, mutant phenotypes, genetic interactions, or expression patterns. All of this information, plus links to additional related data, appears on the gene report pages, accessible via searches from the RGD home page.

**Figure 4:**
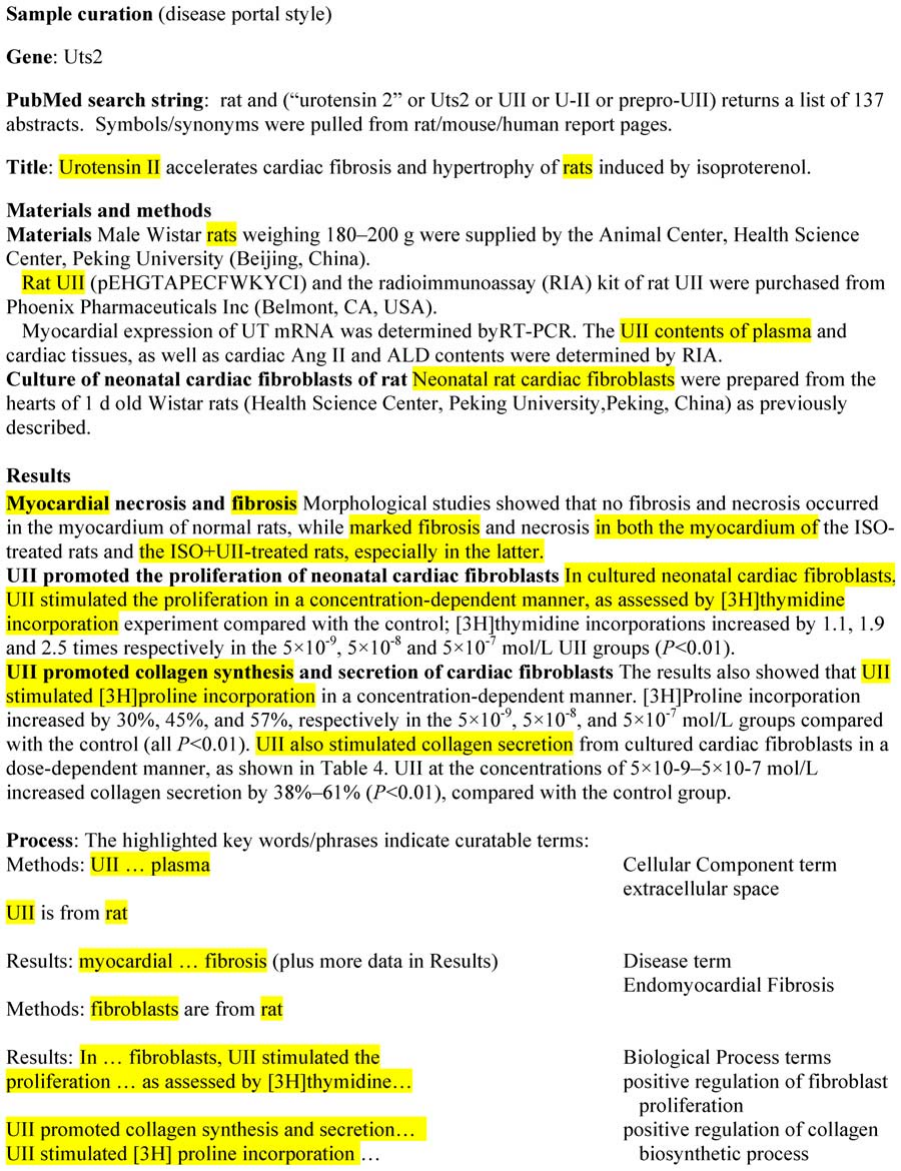
Manuscript curation example. The highlighted manuscript data are annotated as the indicated Gene and Disease ontology terms.

On average the curators at RGD extract information from approximately 5,000 papers per year. From these, multiple types of biological information are annotated for approximately 2,000 genes, 180 rat QTLs, and 200 strains per year. While QTL and strain data are curated as papers are published, RGD targets specific gene datasets for curation. These include genes related to specific disease areas (which generate the most literature), gene families or pathways, as well as those done in conjunction with the Gene Ontology Reference Genomes project [9]. To commence curation, a gene list is created from searches in databases such as GeneCards, the Genetic Association Database, Online Mendelian Inheritance in Man (OMIM), disease and mutation specific databases, and the literature. In a recent initiative, the first step of developing searches and compiling, verifying, and processing the gene list took nearly 7 hours. The list of genes is prioritized and distributed among curators who conduct a PubMed search for rat literature for each gene using symbols and names, aliases, and protein names, as well as those for the human and mouse orthologs. Audits have shown that 75%–85% of papers returned in a search are associated with the targeted gene. A number of papers can be discarded by title and another segment through review of the abstract. The remaining papers are opened and read. Some of these do not result in annotations because the organism was not clearly indicated or the paper did not contain relevant data. An example of the process of curating elements from a manuscript is shown in Figure 4. Using customized software, these manual annotations are added to the report pages for the genes being curated.

### User Interfaces and Software Tools

The RGD curators bring extensive, diverse research backgrounds to the design process for user interfaces and software tools available at the RGD Web site. Curators map out use case scenarios for searches, data analysis tools, and other Web site functions based on the points of view of multiple types of researchers. This allows RGD to create tools and interfaces that fulfill the needs of naïve users as well as seasoned users and bioinformaticians. Curators work closely with the bioinformatics staff to develop the conceptual and functional design of curation software and they also test all new tools and features for usability and accuracy of results before they are made available to the user via links from the RGD home page. In addition, their familiarity with emerging literature, new research techniques, and data types, as well as their interactions with the community often lead to proposals for new data components and tools and new features on existing tools. In other cases, the curator is responsible for the primary design and implementation process. For example, a curator creates and populates the interactive pathway diagrams that RGD is publishing using a content management system and the Ariadne Pathway Studio software. Curators play a major role in the development of all aspects of RGD technology from the home page design to the search functions and accompanying results layouts to the genome tools. To coordinate curation and informatics projects, there is a weekly RGD operations meeting attended by all curators, software developers, the program manager, and co-PIs to address deadlines and priorities. To minimize conflict and ensure timely completion of projects, tracking and documentation is accomplished through Microsoft Project and Mindjet MindManager software. Tool bugs and issues are recorded and tracked through JIRA software. Documentation of requirements, statements of work, and release procedures minimize confusion and conflict between curators and bioinformatics staff.

## Why–Facilitating Research

### Saving the Researcher Time

**Figure 5:**
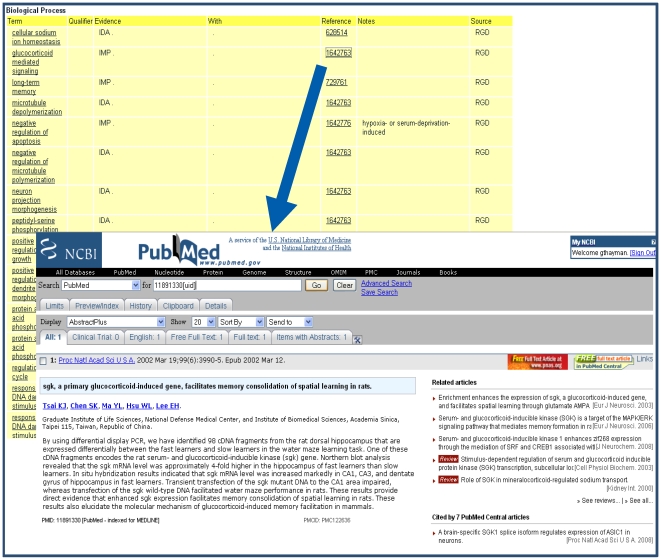
An example of Biological Process annotations with links to references.

To assess the value to researchers of curation work done for genes, RGD undertook two audits. In the first, a review of a subset of cancer genes showed that 3 to 43 papers were curated per gene with an average of 19 papers per gene. In the second study, for a subset of 20 diabetes genes, more comprehensive statistics were tracked. Over 16,000 papers were returned in searches, 410 abstracts reviewed, and 104 full papers read. This produced an average of 20 abstracts and 5 full papers read per gene. Of these, ontology annotations were made from 3–4 papers per gene. Time spent reading abstracts and full papers averaged 106 min per gene. If we extrapolate for the cancer genes in the first study, this would have meant approximately 380 min for each of these more highly published genes. Although three of the targeted genes did not receive any annotations during the process, the remaining 17 received 195 annotations. Because each annotation is linked to the reference from which it was taken (Figure 5), the user can go directly to papers of most interest with little time spent on searches, reviewing titles, or abstracts. As can be seen by the amount of time spent in curation, the time savings for researchers are substantial.

### Education and Outreach

An essential part of the curator's job is to provide education and training for RGD users and potential users. This is accomplished in several ways. The RGD Web site contains a “Help” section developed by the curators and which is accessible from all pages. This component contains a Glossary of Terms, general information on how to use the searches and tools, a Frequently Asked Questions (FAQ) section, and a component that walks users through typical use case scenarios. Curators also handle individual questions through the user request system accessible via the Contact Us button on each page, and through telephone calls and the Rat Community Forum. RGD has published seven tutorial videos at SciVee (http://www.scivee.tv/), a Web site for video publications for research, and videos are available at RGD as well. Curators present tutorials at major conferences such as Experimental Biology, Society of Toxicology, Neuroscience and Rat Genomics and Models, as well as individual rat research laboratories. In addition, RGD is well represented at major conferences such as Biology of Genomes, Genome Informatics, Intelligent Systems for Molecular Biology, and the International Mammalian Genome Conference with presentations and posters highlighting new tools and datasets. Other outreach activities involve contact with individual researchers for nomenclature assignment to genes, QTLs, and strains, as well as construction of customized datasets.

## The Curators' Wish List

The curators at RGD are representative of curators around the world. They are highly trained and experienced researchers dedicated to interpreting and presenting critical information to other researchers via innovative data mining and presentation tools in order to facilitate and enhance their colleagues' work. Their presence and contributions can be seen on RGD Web pages, in the software tools, and in the accurate, well represented data that will lead other researchers to important discoveries. There are, however, a number of developments that would not only make the lives of curators at RGD and around the world better, but improve the curated data they compile and most importantly have a strong, positive impact on all other databases and all researchers at large. Some of these are highlighted here.

### External Requests

1. **Timely access to full text papers.** Even institutions with comprehensive journal subscription programs are not able to provide easy access to every pertinent paper, resulting in delays due to requests for interlibrary loans and other methods used to access these papers. PubMed Central (http://www.pubmedcentral.nih.gov/) currently provides access to full text papers from 450 journals plus those submitted under the NIH Public Access Policy. This policy requires authors funded by NIH to submit full papers published in peer-reviewed journal to PubMed Central within 12 months of publication [10]. While these developments have made access easier, immediate open access at time of publication would allow curators to provide more timely updates to data.
2. **Author submission of data prior to publication.** Those rat researchers who submit data to RGD prior to publication receive RGD IDs for their data and nomenclature review and approval. This ensures that the information in their papers is synchronized with that at RGD and is made available to the public at the time of publication.
3. **Use of unique identifiers in publications.** A time-consuming activity undertaken by curators around the world is verifying identity of genomic data such as genes and QTLs, as well as rat strains. Use of unique identifiers from recognized, international databases and correct nomenclature would make this a much easier task as well as ensure that data are not assigned to the wrong gene, QTL, or strain on the basis of an incorrect identifier used in a publication.
4. **Correct identification of organism used.** As mentioned above, some papers are rejected for curation because the organism used is not identified.
5. **Acknowledgment of their work.** Thousands of researchers worldwide use data from databases in their research on a daily basis. Only a small percentage of them acknowledge in their publications or presentations their use of the data and tools made available through the work of curators. Referencing the sources of the data and tools used in publications would provide recognition of the professional accomplishments of curators just as referencing a colleague's paper provides acknowledgement of the use of his or her work.

### Internal Requests

1. **More sophisticated curation software.** Each development in curation software at RGD has resulted in substantial increases in productivity and accuracy. The next generation software would allow curators to customize interfaces for particular tasks, allowing them to incorporate the necessary functions in ways most useful to them. It would allow them to query multiple databases simultaneously, incorporate sophisticated text mining tools, and run quality control checks at will.
2. **Text mining tools.** Text mining tools are becoming more sophisticated and have gone beyond simple literature search tools. Current work has been focusing on identifying and highlighting ontology terms and other data within the papers. Taking this one step further, an example of a dream tool would be one that takes the annotations that already exist for a gene and matches those against emerging literature to identify novel information, more specific information, or data with better evidence and then would alert the curator to update the gene's information.

Each time researchers access RGD, they are presented with the results of countless hours of work by curators dedicated to providing accurate, up-to-date data and tools to help them make exciting discoveries. Implementing the developments listed above would contribute to increased coverage, comprehensiveness, and ease of use of RGD and databases worldwide.

## Acknowledgments

We wish to thank RGD members Elizabeth A. Worthey, Jeffrey De Pons, Alexander Stoddard, Burcu Bakir Gungor, and Stacy Zacher for their contributions.
